# Using Point-of-Care Ultrasound to Expedite Diagnosis of Necrotizing Fasciitis: A Case Report

**DOI:** 10.21980/J85051

**Published:** 2021-04-19

**Authors:** Romero Kupai, Ashkan Morim, Lucas Friedman, Eva Tovar Hirashima

**Affiliations:** * HCA Healthcare, Riverside Community Hospital, Department of Emergency Medicine, Riverside, CA; ^ University of California Riverside, School of Medicine, Riverside, CA

## Abstract

It is vital that all emergency medicine physicians (EP) are able to perform and evaluate point-of-care ultrasound (POCUS) images when a patient's presentation is concerning for necrotizing fasciitis (NF).

We present a case of a 69-year-old man who had initially presented to an outside hospital with testicular edema and difficulty urinating. Consultative ultrasound was interpreted as a hydrocele and bilateral scrotal pearls but failed to identify the sonographic hallmarks of NF (subcutaneous thickening, free fluid, and subcutaneous air). Computed tomography (CT) performed several hours later re-demonstrated free air and fascial thickening in the scrotum prompting transfer to our hospital for a higher level of care. Upon arrival to our emergency department (ED) POCUS showed extensive free air with shadowing, thickened subcutaneous tissue, and fluid collections. The patient was immediately evaluated by general surgery and urology, who took him to the operating room for debridement.

This case highlights the challenges EP face when assessing patients with NF. Early-on, clinical findings can be subtle and easily missed. POCUS can aid in the diagnosis of this time-dependent disease process. Therefore, it is paramount that EPs look for and recognize characteristic sonographic findings present in NF to improve the accuracy and timeliness of the diagnosis.

**Topics:**

Point-Of-Care Ultrasound, necrotizing fasciitis.

**Figure f1-jetem-6-2-v20:**
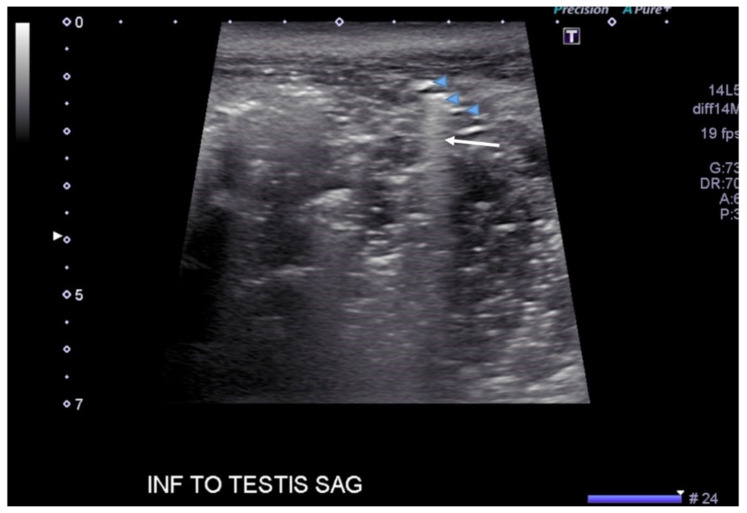


**Figure f2-jetem-6-2-v20:**
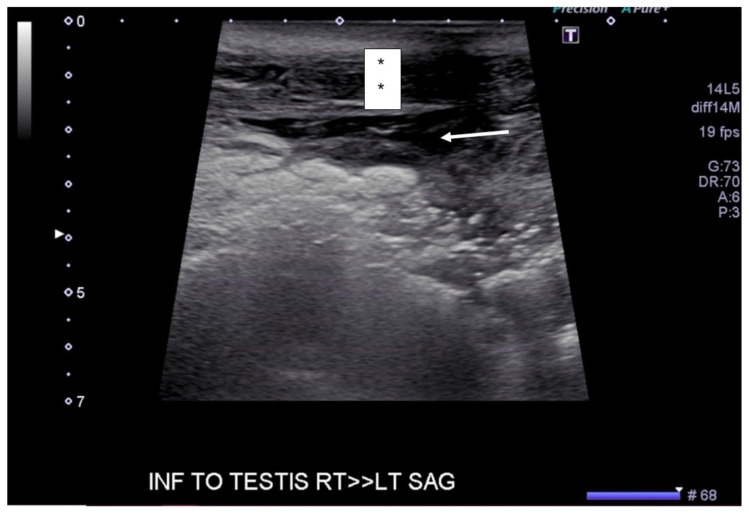


**Figure f3-jetem-6-2-v20:**
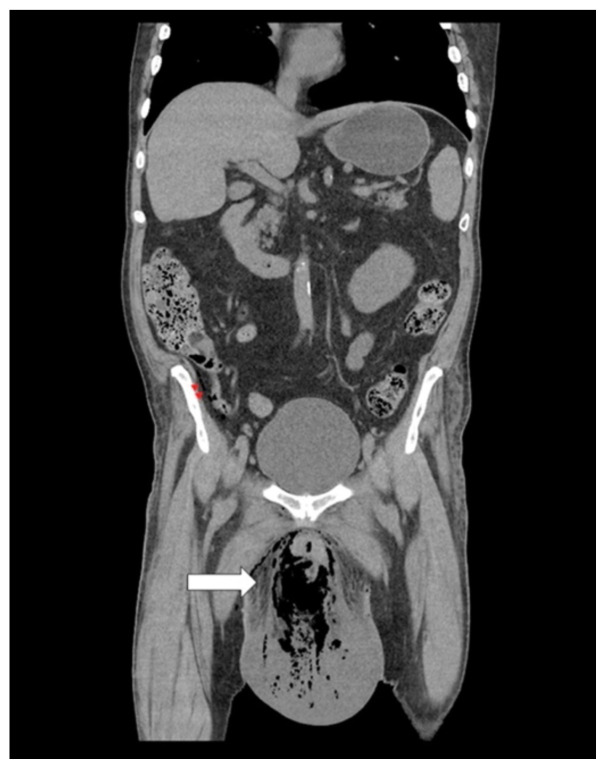


**Figure f4-jetem-6-2-v20:**
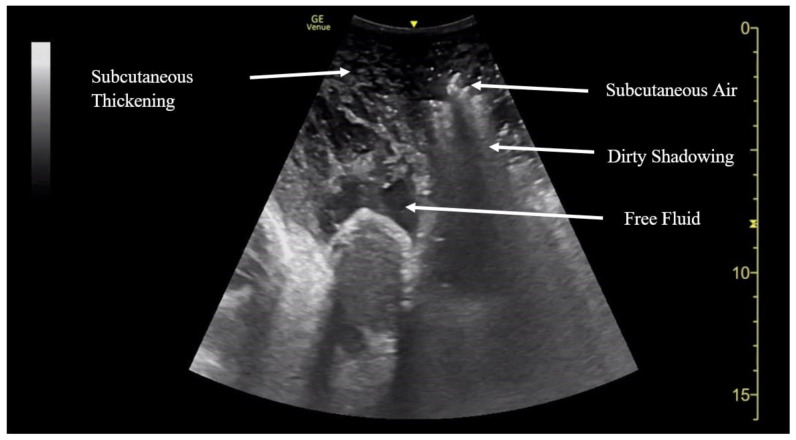
Video Link: https://youtu.be/Se-ELUdqtCk

## Brief introduction

[Fig f1-jetem-6-2-v20][Fig f2-jetem-6-2-v20][Fig f3-jetem-6-2-v20][Fig f4-jetem-6-2-v20]Necrotizing fasciitis is a rapid, devastating disease that results in significant morbidity and mortality if swift intervention is not taken. Historically, mortality has ranged between 25–30%.^1^ Although computed tomography (CT) is currently the gold standard for diagnosis, there is growing evidence that point-of-care ultrasound (POCUS) can aid in the diagnosis, thus accelerating timely surgical management.^2^

## Presenting concerns and clinical findings

A 69-year-old Caucasian male presented to the ED from an outside hospital with suprapubic pain, testicular swelling, and urinary retention. His past medical history was remarkable for type 2 diabetes mellitus (T2D). He was found to be in diabetic ketoacidosis (DKA) with a blood glucose level of 545mg/dL. Additional pertinent labs showed a C-Reactive Protein level of 37.9 mg/dL, sodium 120 mmol/L (corrected 126), creatinine 1.410 mg/dL, and white blood cell count 31.3×10^9 cells/L.

## Significant findings

A consultative scrotal ultrasound was performed, which was read as showing a small right hydrocele, small bilateral scrotal pearls, and normal-appearing testes. Although present, there was no mention of subcutaneous air suggestive of NF, seen in figure 1 as punctate hyperechoic foci (arrowhead) with ring-down artifact known as *dirty shadowing* (arrow). Also, subcutaneous thickening (asterisk) and free fluid (arrow) were seen as shown in figure 2, although their clinical relevance was not recognized in the radiologist's final report. Figure 3 shows an abdominal and pelvic CT that re-demonstrates subcutaneous air in the scrotum and lower abdomen (arrow) as well as fascial thickening of the perineum and free intra-abdominal air. After these images, the patient was transferred to our hospital for further management. Almost immediately after the patient's arrival, POCUS was employed. As seen in figures 4, we were able to identify in just a few minutes extensive subcutaneous air accompanied by *dirty shadowing*, as well as re-demonstration of subcutaneous thickening, fluid collections, and a right hydrocele. Even without the outside hospital's CT, the sonographic findings were highly suggestive for the diagnosis of NF of the perineum, also known as Fournier's gangrene.

## Patient course

The patient was started on clindamycin, ceftriaxone, and vancomycin for NF, as well as an insulin drip for his DKA while at the outside hospital. Upon arrival at our hospital, the patient's vital signs were as follows: blood pressure 106/58, temperature 98.9, heart rate 76 beats per minute, respiratory rate of 18 breaths/minute. The physical examination was remarkable for moderate suprapubic abdominal tenderness with no guarding or rebound. His scrotum was edematous, approximately 12cm by 15cm, with erythema of the scrotal and perineal regions. There were several bullae of various sizes present on the scrotum but no crepitus. The only other notable finding on the physical examination was that he did not seem concerned with his medical condition.

Regarding our ED management, intravenous fluids and insulin were continued, and antibiotic coverage was broadened. Upon careful review of the outside hospital CT, and given the presence of free intraabdominal air, General surgery was also consulted. During his hospital admission, he was taken to the operating room by Urology twice for debridement and stayed in the intensive care unit for management of both NF and DKA. He eventually received a diverting colostomy and was discharged home with home health assistance nine days after the initial evaluation.

## Discussion

Swift diagnosis of NF allows early, aggressive treatment to improve patient outcomes. Our patient had concerning symptoms for NF, including scrotal edema, spreading erythema, and *la belle indifference*.^3^,^4^ He also had several risk factors, most notably his age and immunocompromised state secondary to his uncontrolled T2D. Necrotizing fasciitis is associated with several signs, but these symptoms tend to vary. The most common signs such as erythema, pain out of proportion, and tenderness that goes beyond the erythema, only appear approximately 75% of the time. The more specific findings such as crepitus and skin changes occur even less, with crepitus occurring 50% of the time, and skin changes occurring 38% of the time. Despite extensive free air in the imaging studies, our patient did not have crepitus or skin discoloration. His vital signs were also almost shockingly normal, which was unusual because in a majority of cases with NF, patients are generally febrile and tachycardic.^5^–^7^

Laboratory work can also be useful in diagnosing NF, especially with calculating the laboratory risk indicator for necrotizing fasciitis (LRINEC).^8^ Using several lab results that were listed above, the patient had a LRINEC score of nine. LRINEC scores of six have a positive predictive value of 92.0% for necrotizing soft tissue infections, making the diagnosis of NF very likely. Although LRINEC is a useful tool to help rule-in NF, POCUS is a great tool that can be used to expedite the diagnosis and, more importantly, definitive management.

The STAFF mnemonic (subcutaneous thickening, air, fascial fluid) is a simple way of remembering what needs to be evaluated with POCUS in NF.^9^ Subcutaneous thickening, usually seen early in the disease process, is the least specific of the three findings, given that subcutaneous thickening is also present in cellulitis. In contrast, fascial fluid collections are a specific finding in NF and tend to appear earlier than subcutaneous air.^10^ Additionally, the fluid collection size also correlates with an increasing likelihood of NF, with one cutoff suggesting a fluid collection greater than 2mm.^10^ Of the three sonographic findings, subcutaneous air, seen as punctate hyperechoic foci associated with a ring-down artifact known as *dirty shadowing*, is the most specific. Unfortunately, it occurs late, and thus a clear understanding of all the components of the STAFF mnemonic, as well as the timeline in which each appears, is useful for both radiologist and EP alike. In the case presented, both the outside hospital consultative ultrasound as well as the POCUS performed in our ED revealed all three components of the STAFF mnemonic.

Figures 1 and 2 were obtained with a linear probe, which will result in higher quality images compared to figures 4 which were obtained using a curvilinear probe. The curvilinear probe is beneficial when imaging structures at increased depth. The components of the STAFF mnemonic can be assessed by directly placing the ultrasound probe directly over the areas of concern.

One factor that may have limited the original interpretation done at the outside hospital is that the radiologist only had still images as opposed to video clips. This likely hindered the radiologist from linking any shadowing visualized with the possibility of NF. One advantage of POCUS at the bedside is that the treating physician can interpret the media in a more complete clinical context.

Diagnosing NF quickly is of utmost importance, for as time to definitive treatment increases, so does mortality.^11^ It has been shown that in comparing survivors with non-survivors of NF, the latter had significantly higher times between admission to hospital and definitive surgical treatment.^7^ POCUS is a tool EPs can easily deploy at the bedside to help expedite diagnosis and definitive management.

It should also be noted that according to the American College of Radiology Appropriateness Criteria, they rank x-ray as the ideal imaging modality for suspected NF, with CT imaging ranking as “may be appropriate” and ultrasound as “usually not appropriate.”^12^ This is not consistent with other sources, with CT imaging regarded as the imaging modality of choice if the clinical picture is unclear.^2^,^8^ Ultrasound, especially POCUS, has the added benefit over x-ray in its speed and ease of use at bedside. In addition, x-ray can only visualize subcutaneous emphysema, a late-stage finding, where POCUS can find additional evidence of NF as detailed by the STAFF mnemonic.

Considering the variability in the presentation of NF, POCUS is a useful tool that allows real-time evaluation without requiring the wait times associated with CT. Using the STAFF mnemonic, EP can identify potential NF cases quickly, allowing the early involvement of key surgical consultants. As more EP employ POCUS in their workup of NF, this may lead to quicker diagnosis, and perhaps decreased morbidity and mortality.

## Supplementary Information












