# Supplementary figures and images for: Using Point-of-Care Ultrasound to Expedite Diagnosis of Necrotizing Fasciitis: A Case Report
Source: J Educ Teach Emerg Med. 2021 Apr 19;6(2):V20–4. doi: 10.21980/J85051 (PMC10332782; doi:10.21980/J85051)

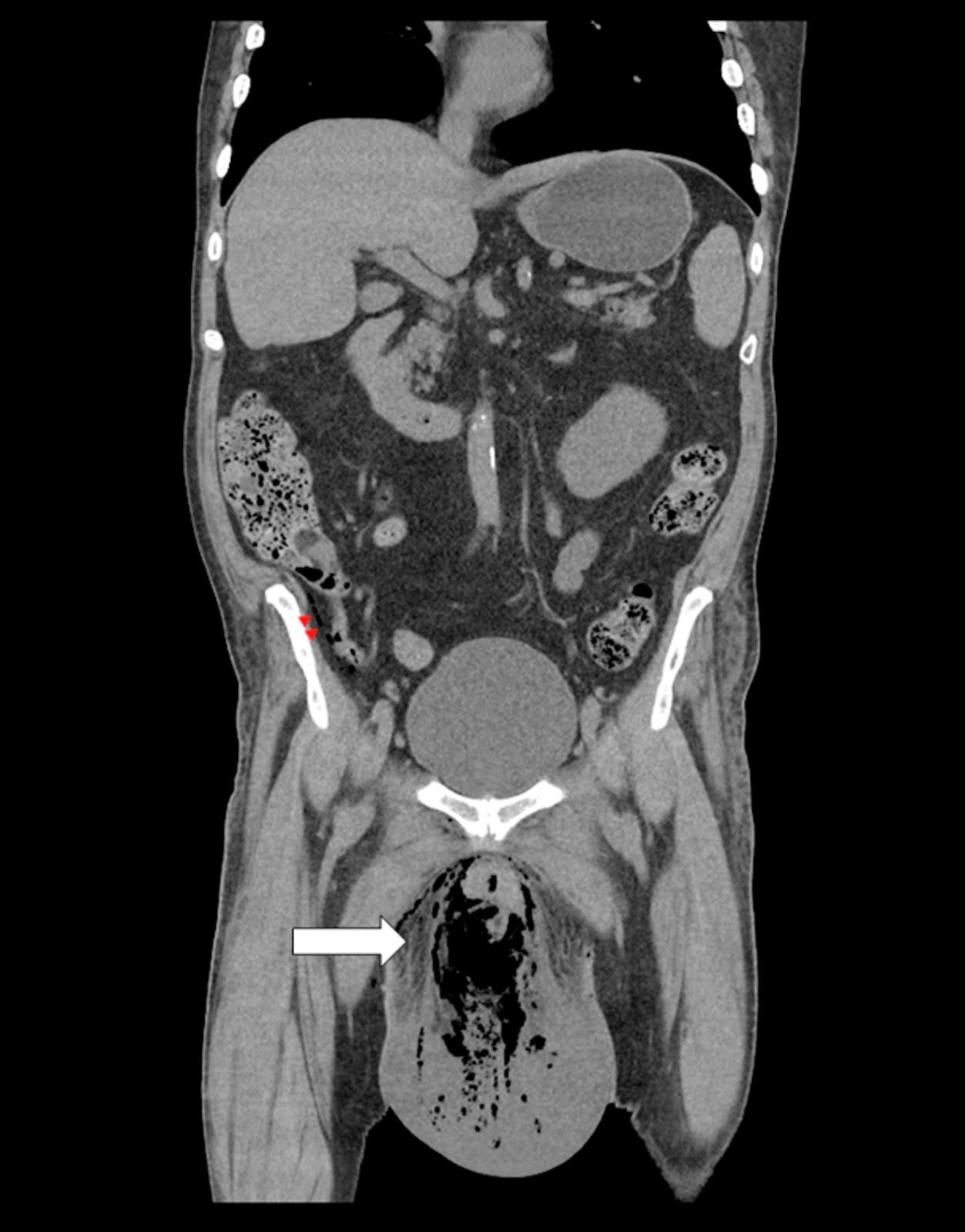

Supplement: Supplementary file 1 [file jetem-6-2-v20-supp1.jpg]

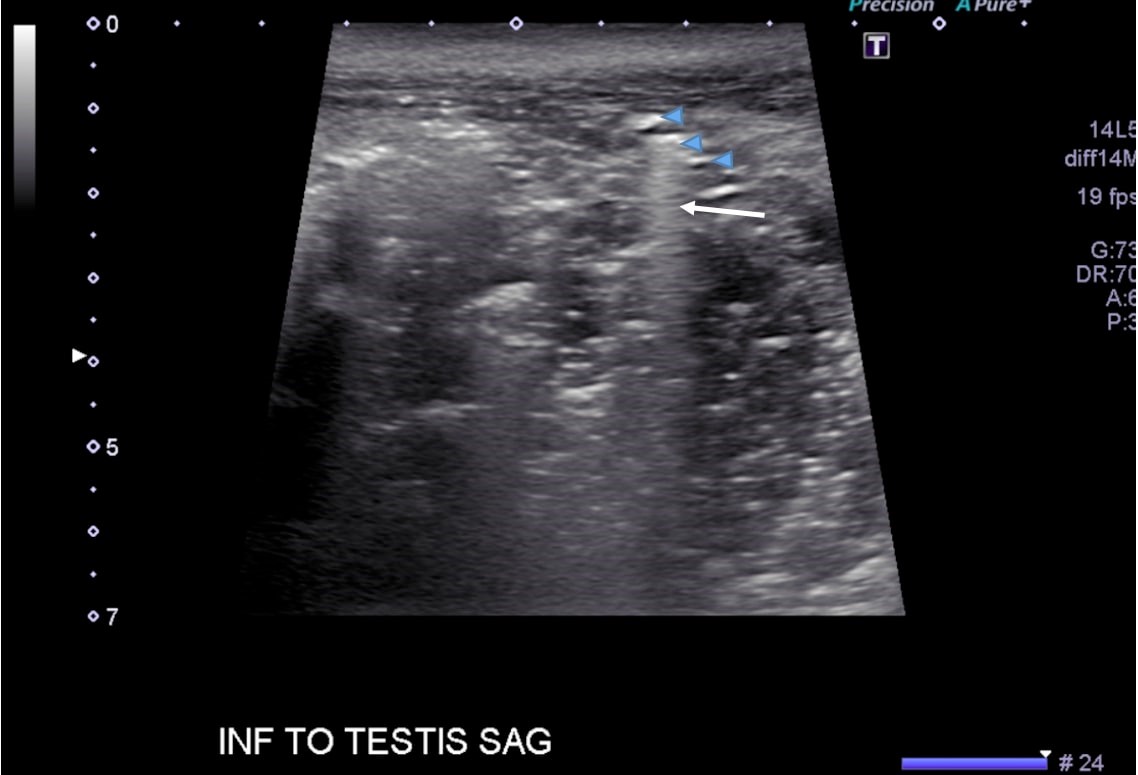

Supplement: Supplementary file 2 [file jetem-6-2-v20-supp2.jpg]

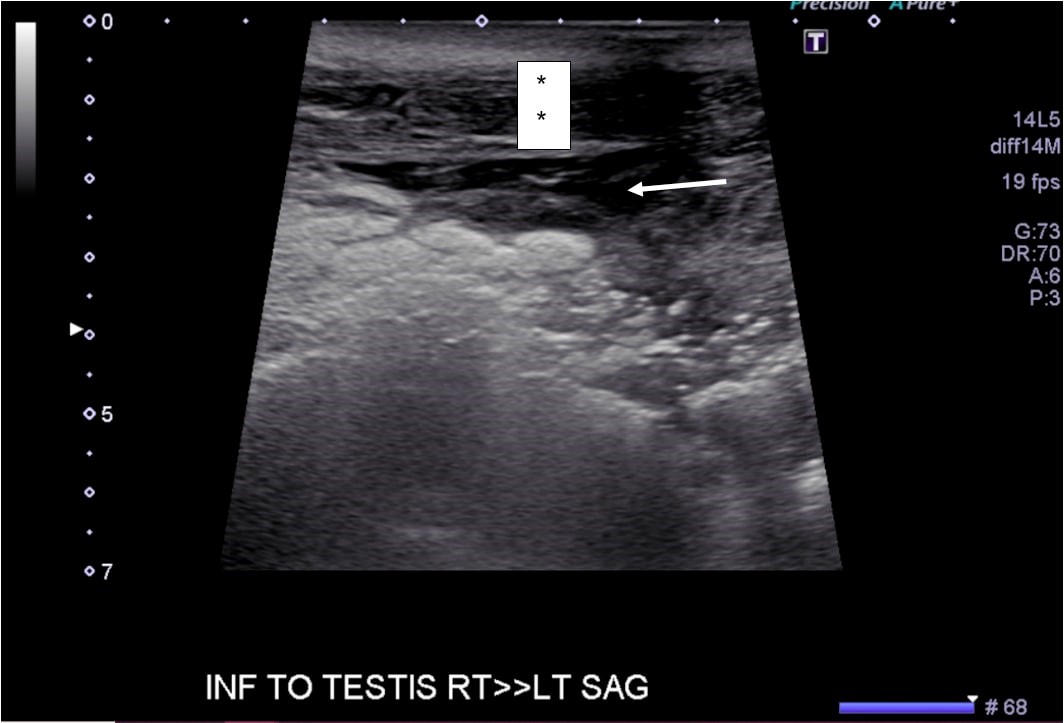

Supplement: Supplementary file 3 [file jetem-6-2-v20-supp3.jpg]

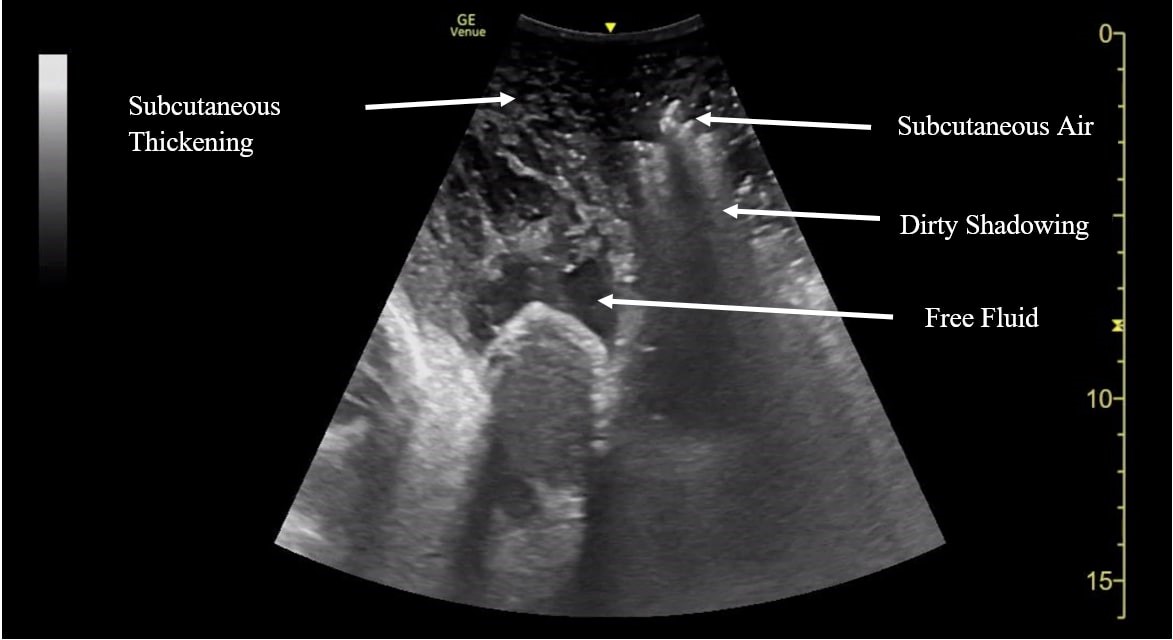

Supplement: Supplementary file 4 [file jetem-6-2-v20-supp4.jpg]

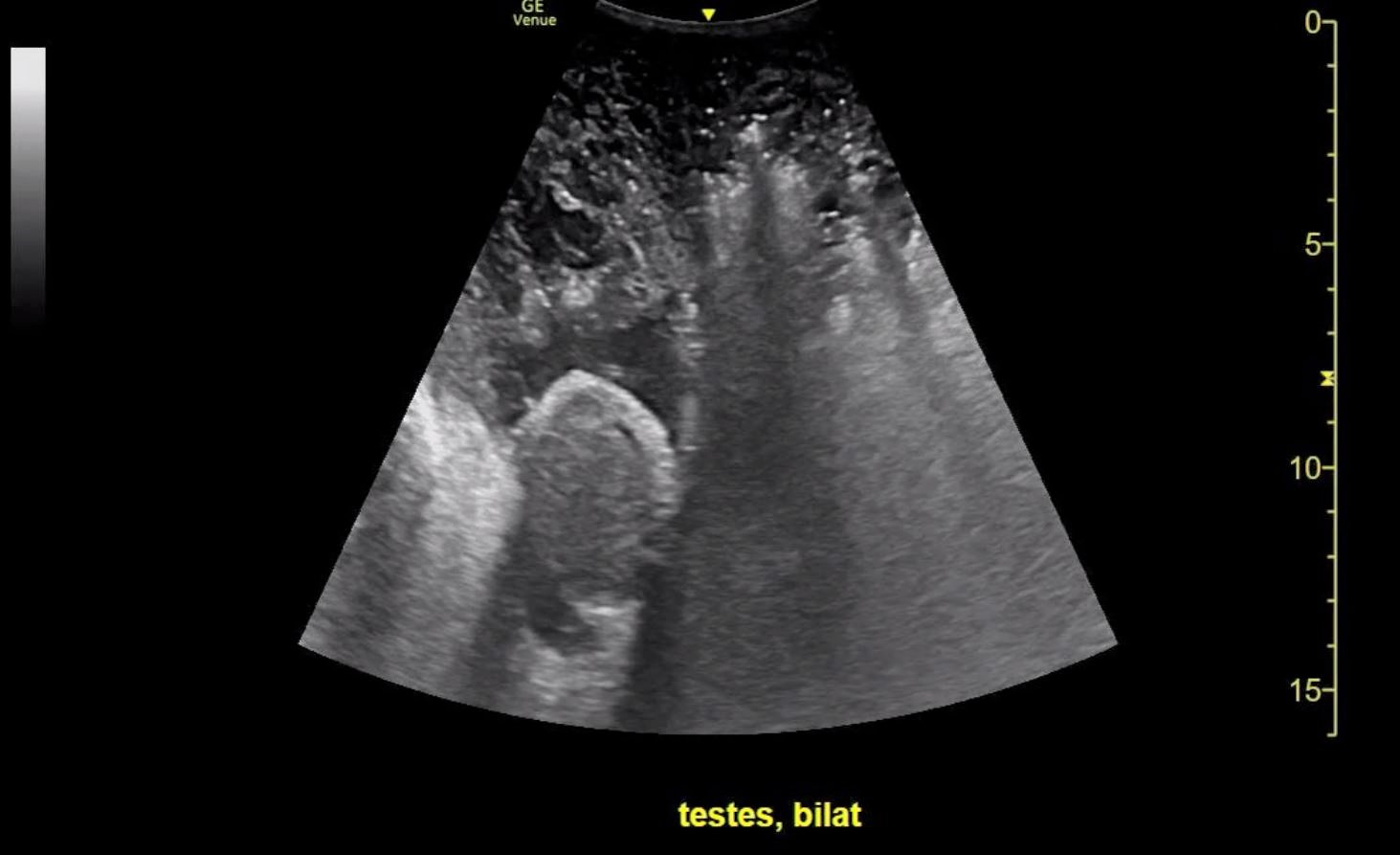

Supplement: Supplementary file 6 [file jetem-6-2-v20-supp6.jpg]
